# Renal infarction in a patient with thyrotoxicosis‐induced atrial fibrillation treated successfully with dabigatran, a case report and literature review

**DOI:** 10.1002/ccr3.6693

**Published:** 2022-12-05

**Authors:** Anas Al‐sadi, Mohammed Abdulgayoom, Mohammed Alamin, Jouhar Kolleri, Israa Jawarneh, Qusai Almaharmeh

**Affiliations:** ^1^ Department of Internal Medicine Hamad Medical Corporation Doha Qatar; ^2^ Department of Radiology Hamad Medical Corporation Doha Qatar; ^3^ Department of Internal Medicine King Abdullah University Hospital Irbid Jordan; ^4^ Department of Internal Medicine Saint Michael's Medical Center Newark New Jersey USA

**Keywords:** atrial fibrillation, dabigatran, direct oral anticoagulation, hyperthyroidism‐induced atrial fibrillation, renal infarction

## Abstract

Renal infarction is an underdiagnosed condition with multiple possible causes, including atrial fibrillation. The treatment approach includes percutaneous endovascular therapy (PET) to restore blood flow, antiplatelet therapy, anticoagulation, or combination therapy, depending on the patient's status and available modalities. Warfarin is the standard anticoagulation therapy, although direct oral anticoagulation (DOAC) therapy is getting more popular. Here, we present a 60‐year‐old male patient with hyperthyroidism complicated by acute renal infarction, which was successfully treated with dabigatran, evident by non‐recurrence and restoration of blood flow in a follow‐up CT angiogram. This case report may open the door for the use of DOAC in acute renal infarction though more studies are needed to prove the efficacy.

## Graphical abstract

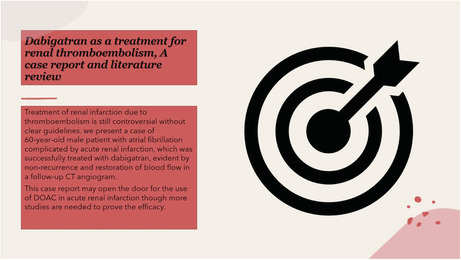

## 1 INTRODUCTION

Renal infarction (RI) is a rare disease that results from disruption of blood flow to the kidneys due to renal artery occlusion. It is usually misdiagnosed or diagnosed late because of its rarity and non‐specific clinical presentation,^1^ which can be easily confused with other more common conditions such as urinary tract infection, nephrolithiasis, and mesenteric ischemia.^2^

Abdominal or flank pain is the usual presenting symptoms in renal infarction; however, asymptomatic incidental finding has been reported. Physical examination and blood tests have low specificity in diagnosis of renal infarction; so, physician need to have low threshold for diagnosis.

The treatment of choice for RI is still controversial due to the lack of randomized clinical trials to compare different approaches. We are presenting a case of thromboembolic RI due to atrial fibrillation who was successfully treated with dabigatran.

## 2 CASE PRESENTATION

A 60‐year‐old male patient with a past medical history of diabetes mellitus type 2, essential hypertension, and hyperthyroidism, presented to the emergency department with severe left flank pain for 2 days. The pain started gradually in the periumbilical area and then localized to the left flank; it was colicky and reached 8 out of 10 on the pain severity scale, associated with a feeling of hotness, sweating, and vomiting.

He described milder attacks of similar pain that happened intermittently from time to time. There was no gross hematuria, or dysuria.

He denied any history of renal stones, and he was not known to have atrial fibrillation.

Regarding his previous history of hyperthyroidism, he was prescribed propranolol and methimazole, but he was not compliant with medications or follow‐up.

On examination, he looked in pain and had high blood pressure of 150/93 mmHg and irregularly irregular pulse at a rate of 110 beats/minute. There was exophthalmos and fine hand tremor, but the thyroid gland was not palpable. His abdomen was soft and lax with mild tenderness in the left lower quadrant and positive left costovertebral angle tenderness. Respiratory, cardiovascular, and neurologic examinations were unremarkable.

**FIGURE 1:**
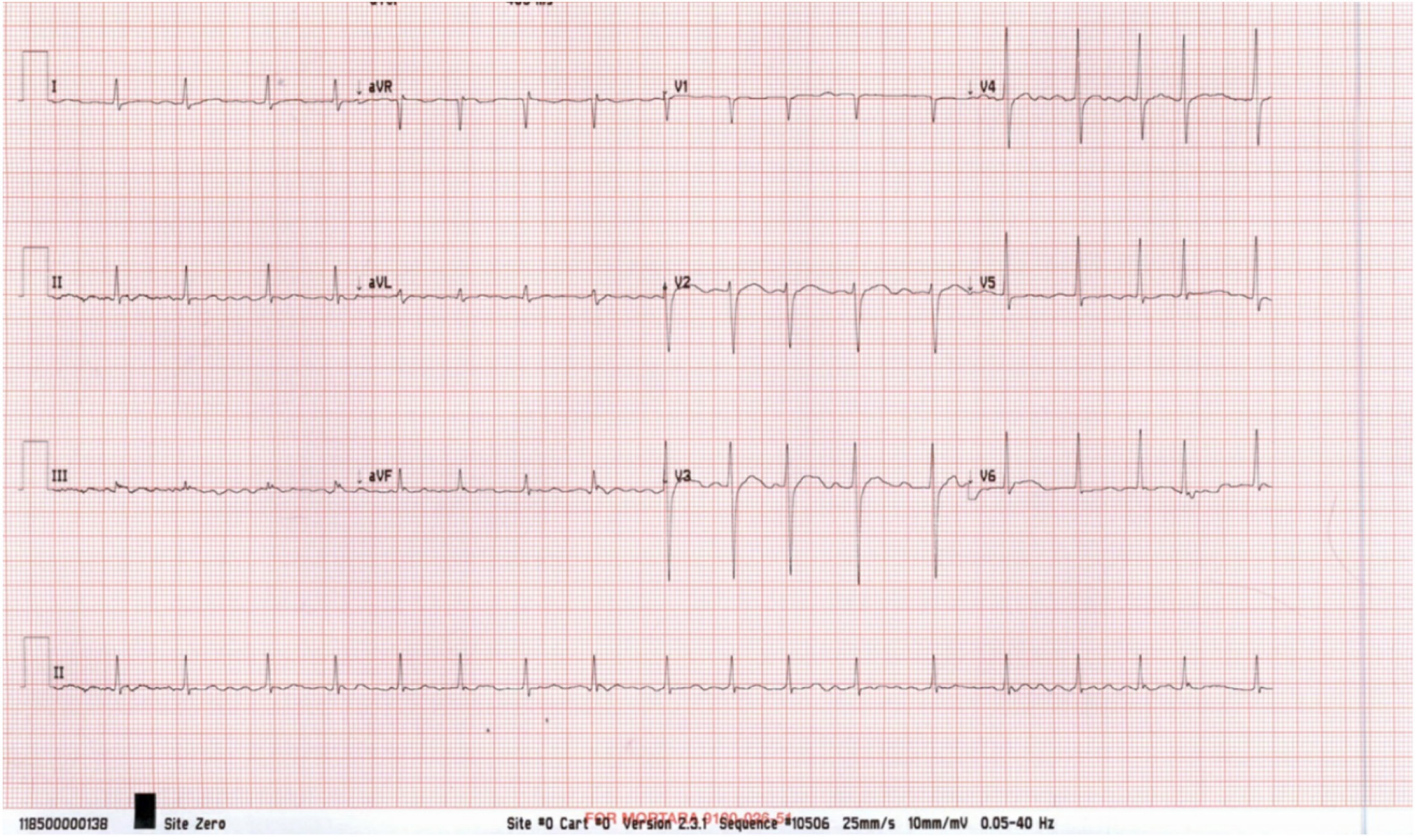
ECG showed atrial fibrillation with a ventricular rate around 100 beats/min.

His blood works (Table 1) were remarkable for marked leukocytosis, icroscopic hematuria, severe hyperthyroidism, and mild renal impairment, which normalized over a few days. ECG was done and confirmed the presence of atrial fibrillation (Figure 1).

**TABLE 1:** Significant initial investigations

White blood cell	25,000/μl (9200/μl after 5 days)	INR	1.1
Hemoglobin	15.9 g/dl	PTT	33 s
Platelet	200,000/μl	Bicarbonate	21 mmol/L
Creatinine	118 μmol/L (89 μmol/L after 5 days)	HbA1c	6.0%
Urea	7.8 mmol/L	ALT	15 U/L
Sodium	133 mmol/L	AST	22 U/L
Potassium	4.3 mmol/L	TSH	<0.01 mIU/L
Urine RBC	55 μl	FT4	68.6 pmol/L

**FIGURE 2:**
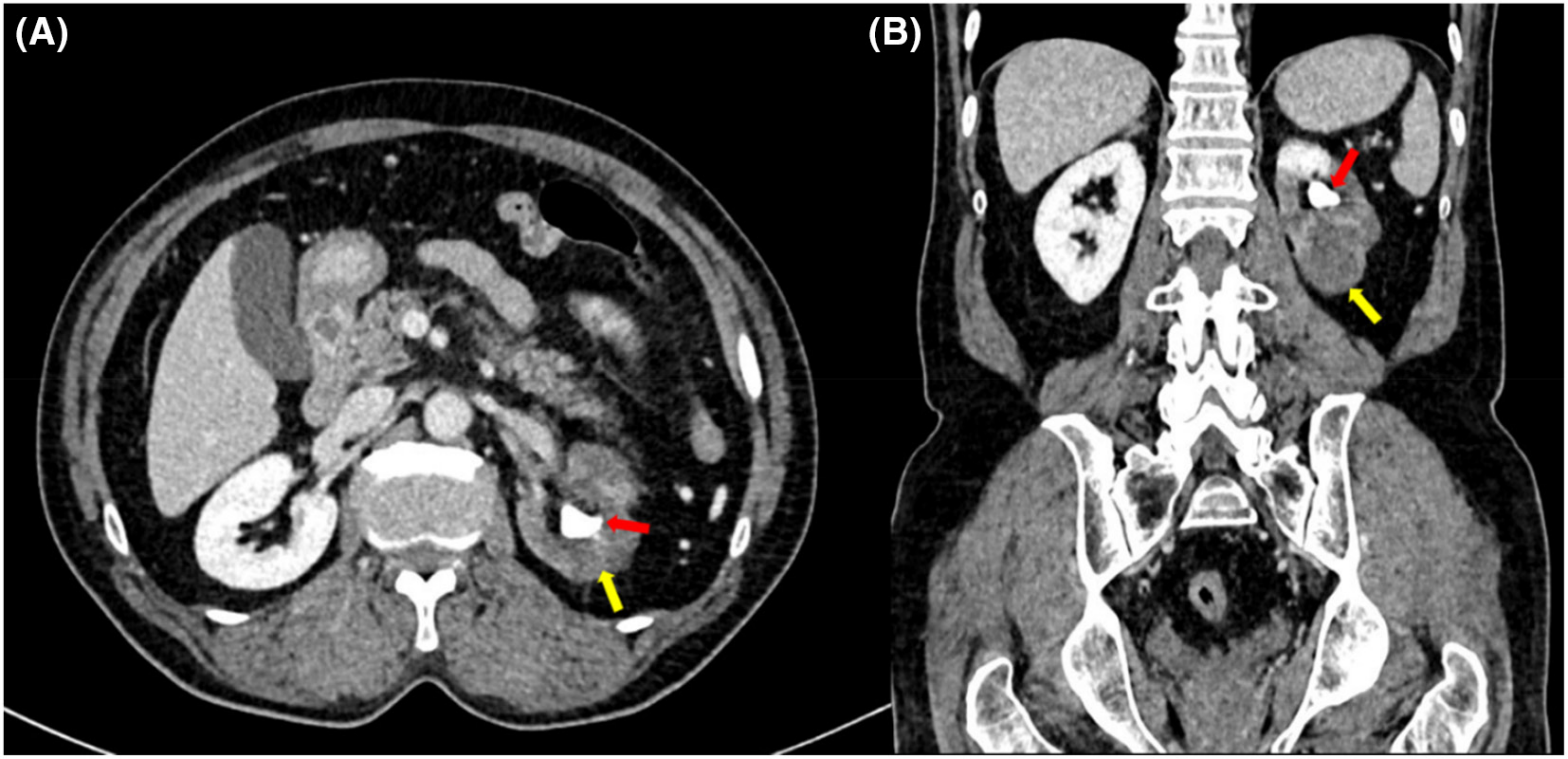
CT abdomen and pelvis with IV contrast (A) Axial and (B) Coronal cuts showing inhomogeneous parenchymal enhancement of left kidney with no excretion (Yellow arrows). A 16 mm calculus is seen in the left renal pelvis (Red arrows).

Urgent CT abdomen and pelvis with intravenous contrast was done and showed inhomogeneous parenchymal enhancement of the left kidney with no excretion seen. There was a 16 mm non‐obstructing stone in the left renal pelvis. No back pressure was seen (Figure 2).

**FIGURE 3:**
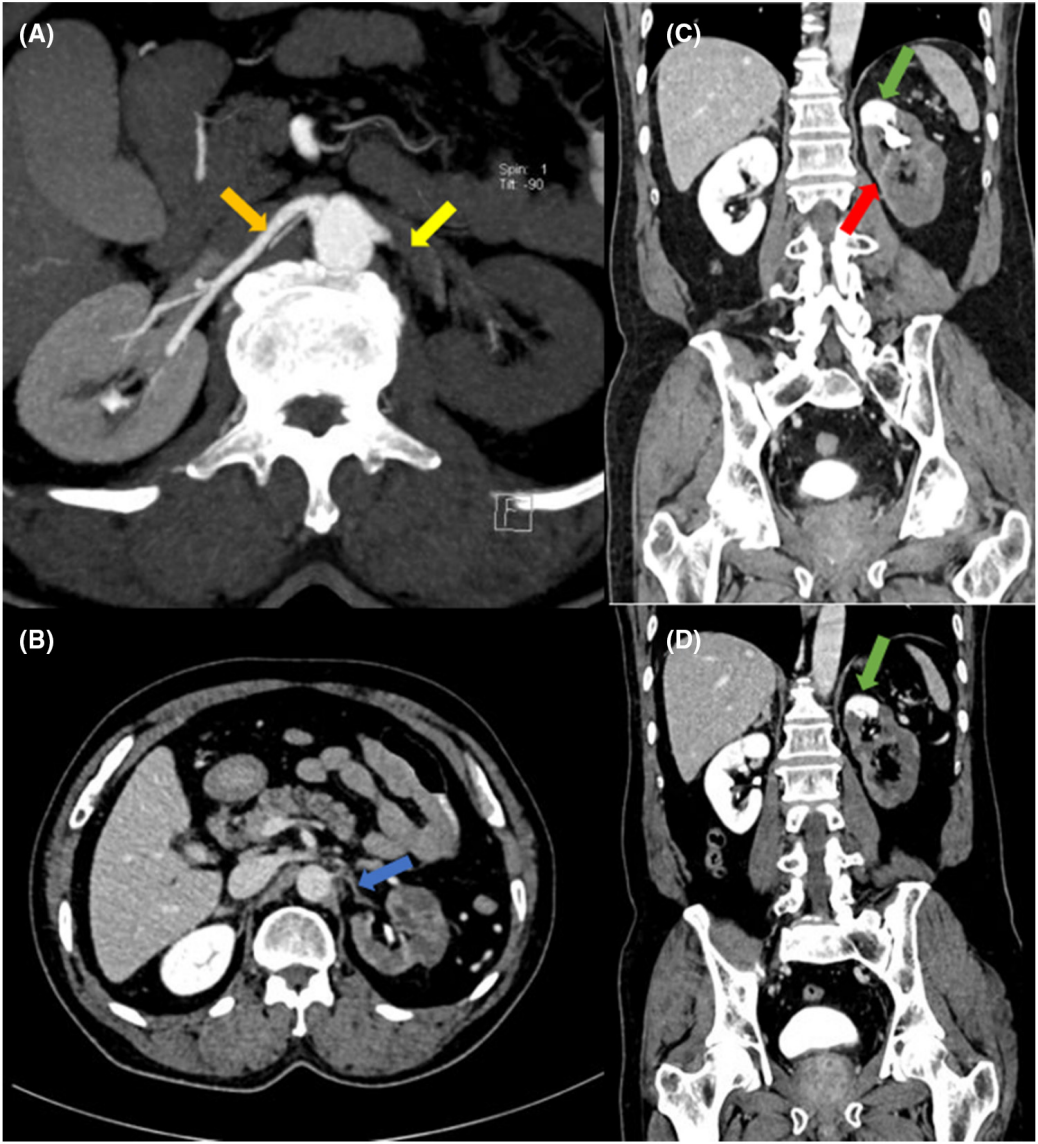
CT angiogram of renal arteries (A) Axial maximum intensity projection, (B) Axial and (C, D) Coronal cuts showing non‐opacification of left main renal artery just beyond its origin (Yellow arrow) with hypoperfusion of left kidney, and faint peripheral enhancement of left kidney (Red arrow). An accessory left renal artery was seen arising from the aorta (Blue arrow) supplying the upper pole of left kidney (Green arrows). The right renal artery is patent (Orange arrow).

An emergency CT angiogram of renal arteries was performed and revealed non‐opacification of the left main renal artery just beyond its origin with subsequent hypoperfusion of the left kidney. Faint peripheral enhancement of the left renal artery was noted in the venous phase. An accessory left renal artery was noted arising from the aorta supplying the upper pole and perfusing the upper pole of left kidney. The right renal artery was patent, and the right kidney showed good cortical enhancement (Figure 3). These findings gave an impression of near total occlusion of the left main renal artery.

He was commenced immediately on heparin infusion, and angioplasty confirmed acute total occlusion of the left renal artery; unfortunately, mechanical thrombectomy, and local thrombolysis failed. During the procedure, the vascular surgery team was also involved, but no intervention was applicable from their side, so medical treatment of the thrombus was the only suitable option. Dabigatran was chosen over warfarin as the patient is working far away and will not be able to monitor INR level regularly.

**FIGURE 4:**
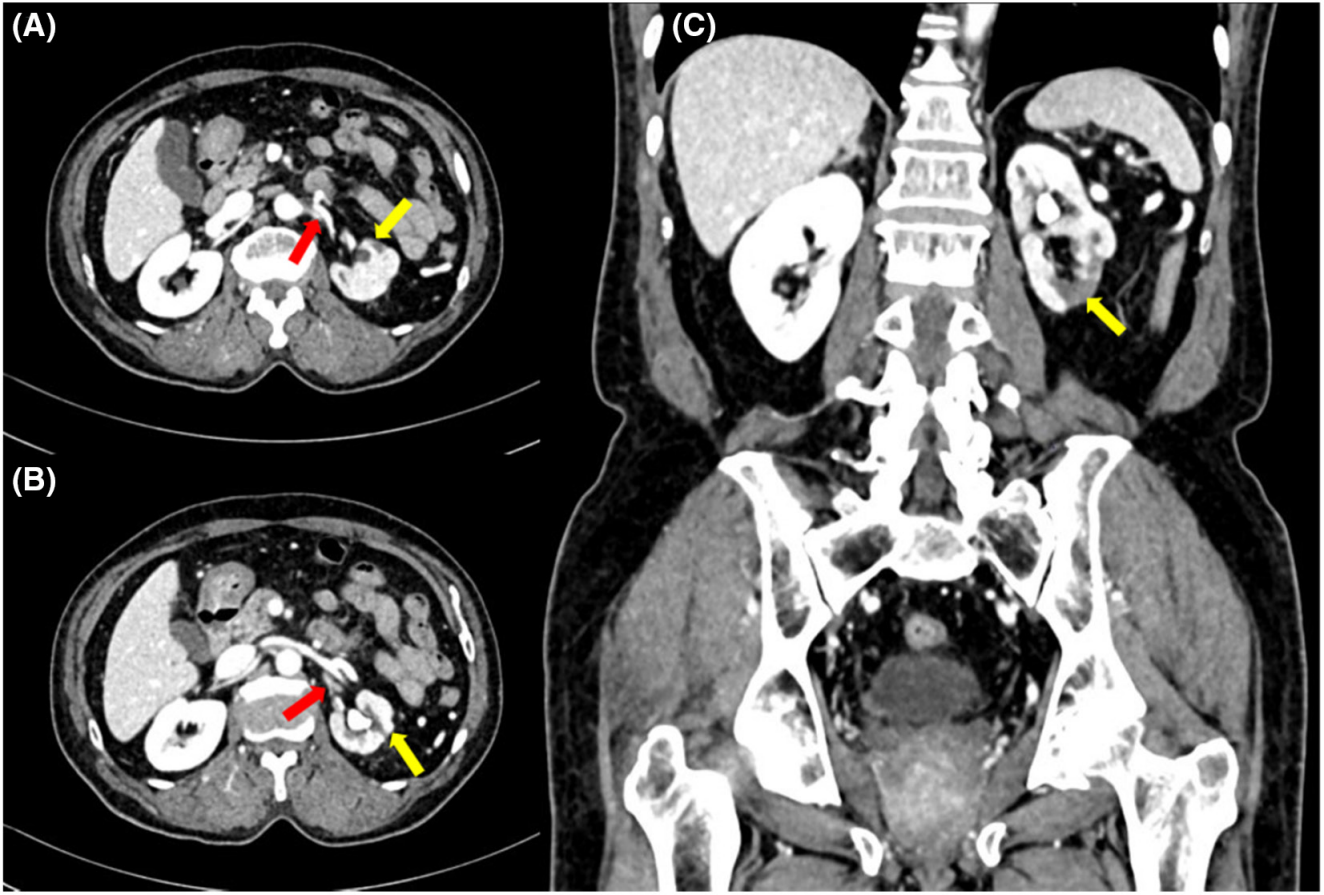
CT abdomen and pelvis with IV contrast (A, B) Axial and (C) Coronal cuts showing decreased caliber of left main renal artery (Red arrows). Multiple hypodense non enhancing areas are seen in the left kidney (Yellow arrows).

Four months later, he presented again to the emergency department with generalized abdominal pain; He underwent an urgent CT abdomen with contrast to rule out mesenteric blockage. The scan did not show definite evidence of mesenteric vascular occlusion. However, the left renal artery was smaller than in the previous scan, along with preserved flow(which indicates the resolution of the previously mentioned thrombus) (Figure 4). The left kidney was smaller, with multiple hypodense areas representing prior infarcts. The patient was discharged on his last home medications, including Dabigatran.

## 3 DISCUSSION

Actual incidence of renal infarction is not precisely known. One autopsy study in 1940 estimated the incidence of RI at 1.4%^3^; however, incidence rate in another study in 2006 was found to be at 0.3%.^4^ Atrial fibrillation is the most common underlying etiology and was identified as the primary causal factor in 64% of published cases of RI.^5^ The reported incidence of renal thromboembolism in patients with atrial fibrillation was 2% in a case series of 30,000 patients followed for 13 years.^6^ Other etiologies include endocarditis, hypercoagulable disorders, hematologic disease, and trauma.^1^, ^7^ It is well known that hyperthyroidism for any reason shift hemostasis toward prothrombotic state.^8^

The usual presenting symptom in patients with RI due to renal artery embolism is abdominal or flank pain which is present in more than 50% of patients.^1^ Other common symptoms are nausea, vomiting, and sometimes fever,^9^ whereas asymptomatic incidental findings also have been reported. Elevated blood pressure at the time of presentation occurs in almost half of patients with RI.^1^ Patients typically have elevated white cell count, lactate dehydrogenase, and serum creatinine.^10^ High serum lactate dehydrogenase is considered a sensitive marker for RI; however, it lacks specificity. Elevated serum creatinine levels correlated with longer hospitalization length.^10^

Giving its rarity and non‐specific symptoms and signs, diagnosis of RI is challenging. A physician should keep a low threshold for diagnosis of RI as diagnoses are often delayed,^11^ with fewer than 50% of cases being diagnosed promptly.^2^ The gold standard for diagnosing renal artery embolism and RI is angiography; however, other imaging modalities are helpful. The diagnostic test of RI in the course of atrial fibrillation was evaluated in a case series of 44 patients. Renal isotope scan detected 97% of cases, while CT with contrast enhancement and ultrasound detected 80% and 11%, respectively.^2^ Angiography was positive in all cases.^5^ Contrast‐enhanced CT scans facilitate a fast, non‐invasive way of diagnosis.^12^ In our patient, CT angiography showed non‐opacification of the left main renal artery just beyond its origin with subsequent hypoperfusion of the left kidney. Follow‐up CT was remarkable for hypodensities that represent renal infarction with restoration of blood flow after few months of treatment with dabigatran. One reported specific sign for renal infarction in contrast‐enhanced CT scan is the cortical rim sign which is seen in almost 50% of patients with RI and results from intact collateral vascular circulation.^13^

Among 47 patients with acute renal embolism who followed for 41 months, only one patient required dialysis.^14^ However, patients with renal infarction have an all‐cause mortality rate of 19.7% at 40 months.^15^

The treatment modality of choice for renal artery embolism is controversial as the superiority of a particular therapy has not been evaluated in randomized clinical trials. Reported approaches include anticoagulation, percutaneous endovascular treatment such as thrombolysis, thrombectomy with or without angioplasty or stent placement, and open surgery. Most experts usually prefer restoration of renal blood flow with endovascular approaches. However, the main limitations are the availability of well‐trained interventional radiologists with good experience in treating renal artery thromboembolism as well as procedural complications. Systemic thrombolysis using a standard dose commonly used for myocardial infarction and pulmonary embolism also has been reported as a successful treatment for renal artery thromboembolism^15^ but with a higher risk of systemic complications. The maximum duration of complete renal artery occlusion after which thrombolysis will not be beneficial is unknown. However, one case report describes successful thrombolysis after 48 hours of the onset of symptoms.^16^

In our patient, embolectomy was attempted first but unfortunately failed. Then the patient was kept on anticoagulation. Dabigatran was chosen over warfarin as the patient is working in a faraway and will not be able to monitor INR level.

Follow‐up CT scan 3 months later showed successful recanalization while kidney function had not been affected. Dabigatran, among other DOAC, has been approved for prevention of stroke and systemic thromboembolism in patients with atrial fibrillation^17^, ^18^; however, the role and efficacy of direct oral anticoagulations in treating renal artery thromboembolism is not studied and up to our knowledge this is the first case that describe successful treatment of renal thromboembolism with dabigatran without radiological intervention. Our case may suggest a promising role requiring further evaluation in randomized clinical trials.

## 4 CONCLUSION

Treatment of renal infarction due to thromboembolism is still controversial without clear guidelines. However, immediate restoration of blood flow by endovascular approach should be attempted when possible. Using dabigatran as an alternative treatment to warfarin has a promising outcome. Further reporting and clinical trials are needed to establish clear guidelines for optimum anticoagulation.

## AUTHOR CONTRIBUTIONS

**Anas Al‐sadi:** Data curation; funding acquisition; resources; writing – original draft. **Mohammed Abdulgayoom:** Software; supervision; writing – original draft. **Mohammed Alamin:** Validation; writing – original draft. **jouhar kolleri:** Investigation; software. **Israa Jawarneh:** Methodology; visualization; writing – original draft; writing – review and editing. **Qusai Maharmeh:** Supervision; writing – review and editing.

## CONFLICT OF INTEREST

No conflict of interest to be declared.

## CONSENT

Written informed consent was obtained from the patient to publish this report in accordance with the journal's patient consent policy.

## Data Availability

None.
